# Tripartite motif-containing 3 (TRIM3) enhances ER signaling and confers tamoxifen resistance in breast cancer

**DOI:** 10.1038/s41389-021-00350-x

**Published:** 2021-09-10

**Authors:** Runyi Ye, NiJiati AiErken, Xiaying Kuang, Huijuan Zeng, Nan Shao, Ying Lin, Pian Liu, Shenming Wang

**Affiliations:** 1 Department of Thyroid and Breast Surgery, The First Affiliated Hospital of Sun Yat-sen University, Guangzhou, Guangdong China; 2 Department of Breast and Thyroid Surgery, The Seventh Affiliated Hospital, Sun Yat-sen University, ShenZhen, 518107 China; 3 Cancer Center, Union Hospital, Tongji Medical College, Huazhong University of Science and Technology, Wuhan, 430022 China

**Keywords:** Breast cancer, Prognostic markers

## Abstract

Tamoxifen resistance remains a clinical problem in estrogen receptor (ER)-positive breast cancer. SUMOylation of ERα enhances ERα-induced transcription activity. Tripartite motif-containing (TRIM) proteins are a new class of SUMO E3 ligases, which regulate the SUMOylation of proteins. However, the precise molecular mechanism and function of TRIM3 in SUMOylation and the response to tamoxifen remain unclear. In the present study, we observed that TRIM3 was dramatically overexpressed in breast cancer, which correlated with tamoxifen resistance. Furthermore, TRIM3 overexpression significantly correlated with poor survival of patients with ER^+^ breast cancer treated with tamoxifen. TRIM3 overexpression conferred cell survival and tumorigenesis, whereas knocking down of TRIM3 reduced these capabilities. Moreover, TRIM3, as a ubiquitin carrier protein 9 (UBC9) binding protein, promoted SUMO modification of estrogen receptor 1 (ESR1) and activated the ER pathway. Silencing UBC9 abolished the function of TRIM3 in regulating tamoxifen resistance. These results suggest TRIM3 as a novel biomarker for breast cancer therapy, indicating that inhibiting TRIM3 combined with tamoxifen might provide a potential treatment for breast cancer.

## Introduction

Representing almost 25% of cancer cases among women, breast cancer remains a global challenge worldwide [1]. Estrogen receptor alpha (ERα) is the main molecular target for endocrine therapies, which antagonize the ER and suppress estrogen synthesis to inhibit tumor growth [2]. Tamoxifen, as an adjuvant endocrine therapy, is widely used as an ER antagonist in breast cancer, which blocks the binding between estrogen and the ER, and suppresses ERα target genes [3]. Unfortunately, ~30–40% of patients with ER^+^ breast cancer fail to respond (de novo resistance) or become resistant (acquired resistance) to tamoxifen, with a deadly outcome, which presents a major clinical challenge in the treatment of breast cancer [4, 5]. Therefore, it is important to determine the underlying molecular mechanism of endocrine resistance and to develop novel potential therapies for breast cancer.

Evidence has proved that several mechanisms contribute to tamoxifen resistance, such as activation of receptor tyrosine kinases (RTK) signal transduction pathways, activation of oncogenic signaling pathways (e.g., the phosphatidylinositol-4,5-bisphosphate 3-kinase (PI3K)/protein kinase B (Akt)/mammalian target of rapamycin (mTOR) pathway, and the nuclear factor kappa B (NF-κB) pathway) and modulation of ER signaling [6–9]. The ER is widely expressed in breast cancer (almost 70%), which is a strong predictor for tamoxifen therapy [10]. Notably, loss of estrogen receptor 1 (ESR1) expression confers tamoxifen resistance, which is reported in 15–20% of breast cancer cases [10, 11]. However, the ER in most tamoxifen-resistant cases remains to be expressed and activated [10], suggesting that there might other mechanisms that regulate ESR1 to confer tamoxifen resistance. Emerging evidence shows that *ESR1* expression is regulated by diverse aspects, including histone modification [12], DNA methylation [13], somatic mutation [10], *ESR1* fusion genes [14], and post-translational modifications (PTMs) [15]. SUMOylation, as an important PTM, effects subcellular localization, protein–protein interaction, protein stability, and transcriptional activity, and is regulated by three important enzymes: Activating enzyme (E1), conjugating enzyme (E2), and ligases (E3). Sentis et al. proved that small ubiquitin-like modifier (SUMO)-1 modifies ERα via SUMOylation, which enhances ERα-induced transcription activity via improving ERα’s DNA-binding property [16]. However, the mechanism and biological function of ESR1 SUMOylation in breast cancer remain unclear.

Tripartite motif-containing (TRIM) proteins belong to the RING-type E3 ubiquitin ligase family, which are involved cellular signaling, cell growth, and tumorigenesis [17]. Furthermore, TRIM proteins are a new class of SUMO E3 ligases, transferring SUMO to substrates [17, 18]. TRIM27, TRIM32, and TRIM36 are well-known SUMO E3 ligases, which modify SUMOylation via binding to ubiquitin-conjugating enzyme 9 (UBC9) [17, 18]. The gene encoding TRIM3 is located on human chromosome 11p15, which is thought to harbor tumor suppressor genes [19–21]. However, the precise molecular mechanism and function of TRIM3 in SUMOylation and the response to tamoxifen remain unclear.

In the present study, we found that TRIM3 was significantly upregulated in tamoxifen-resistant breast cancer, and was associated with poor survival of patients with breast cancer during tamoxifen therapy. Overexpression of TRIM3 conferred estrogen-independent growth and contributed to tamoxifen resistance. Experiments revealed that TRIM3 upregulated ER SUMO modification and activated the ER signaling pathway by binding to UBC9, which could be abolished by the deSUMOylation enzyme SUMO specific peptidase 1 (SENP1). Taken together, our results revealed the crucial role of TRIM3 in SUMOylation of ESR1 and the modulation of the tamoxifen response, identifying TRIM3 as a potential target to improve clinical outcomes of breast cancer.

## Results

### TRIM3 contributes tamoxifen-resistant in breast cancer

**Fig. 1:**
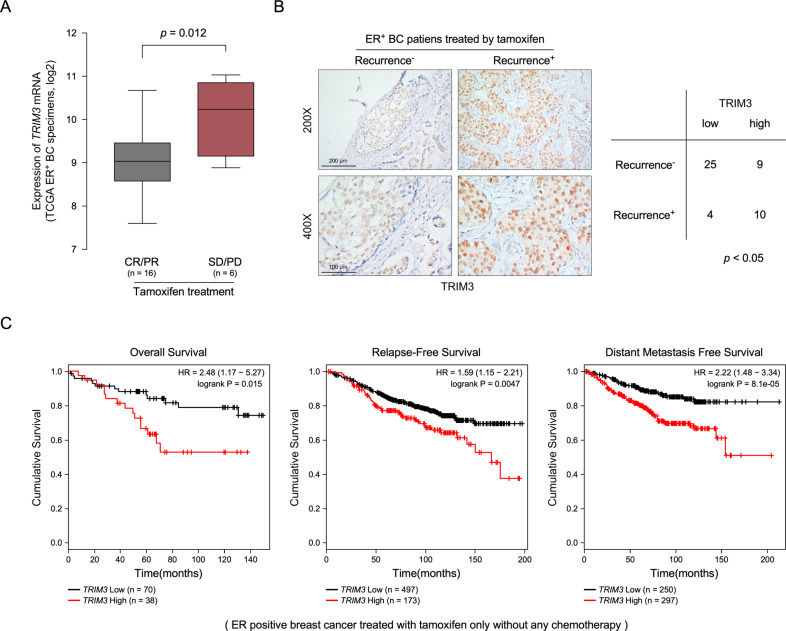
TRIM3 overexpression promotes tamoxifen resistance in breast cancer. **A** A boxplot representing *TRIM3* expression levels in ER^+^ breast cancer with tamoxifen treatment [SD/PD versus CR/PR, *P* = 0.012, stable disease (SD), progressive disease (PD), complete remission (CR), partial remission (PR)]. **B** Representative images of TRIM3 expression in recurrence^−^ and recurrence^+^ ER^+^ breast cancer tissues with tamoxifen treatment (left panel). Scale bars: 200 μm (upper panel) and 100 μm (lower panel). The statistical significance of the TRIM3 levels is assessed in the right panel. *P* < 0.05. **C** Analysis of the effect of *TRIM3* expression on Overall Survival (OS), Relapse-Free Survival (RFS), and Distant Metastasis-Free survival (DMFS) of ER^+^ breast cancer with tamoxifen therapy.

To determine the molecular mechanism that contributes to breast cancer tamoxifen resistance, we analyzed gene expression in patients with breast cancer from TCGA database. As shown in Fig. 1A, the expression of *TRIM3* was significantly higher in patients without a response to tamoxifen therapy [stable disease (SD), progressive disease (PD)] than those who responded to tamoxifen treatment [complete remission (CR), partial remission (PR)]. *TRIM3* mRNA showed higher expression in ER^+^ breast cancer than in ER^−^ breast cancer (Fig. S1A, *p* = 1.365E−68). Furthermore, 48 ER^+^ clinical breast cancer specimens after tamoxifen treatment were used to examine the expression of TRIM3 protein (Table S1). As expected, TRIM3 expression was significantly higher in tamoxifen-resistant ER^+^ breast cancer than that in tamoxifen-sensitive ER^+^ breast cancer (Fig. 1B). Statistical analysis revealed that the expression of *TRIM3* correlated closely with cancer recurrence of patients under tamoxifen treatment (Fig. 1C). By analyzing published expression profiles obtained from breast cancer (TCGA) data, we found that levels of *TRIM3* mRNA correlated positively with levels of the TRIM3 protein, and *TRIM3* mRNA expression was upregulated in ER^+^ breast cancer tissues compared with that in ER^−^ cancer tissues and normal breast tissue (Fig. S1A, B). Consistently, Kaplan–Meier plotter showed that higher expression of *TRIM3* correlated positively with poorer overall survival (OS), relapse-free survival (RFS), and distant metastasis-free survival (DMFS) in cases of ER^+^ breast cancer with tamoxifen treatment and/or chemotherapy, but not in ER^−^ breast cancer (Figs. 1C and S1C). These results revealed that TRIM3 might be associated with the tamoxifen resistance of ER^+^ breast cancer.

### TRIM3 promotes tamoxifen resistance in ER^+^ breast cancer in vitro

**Fig. 2:**
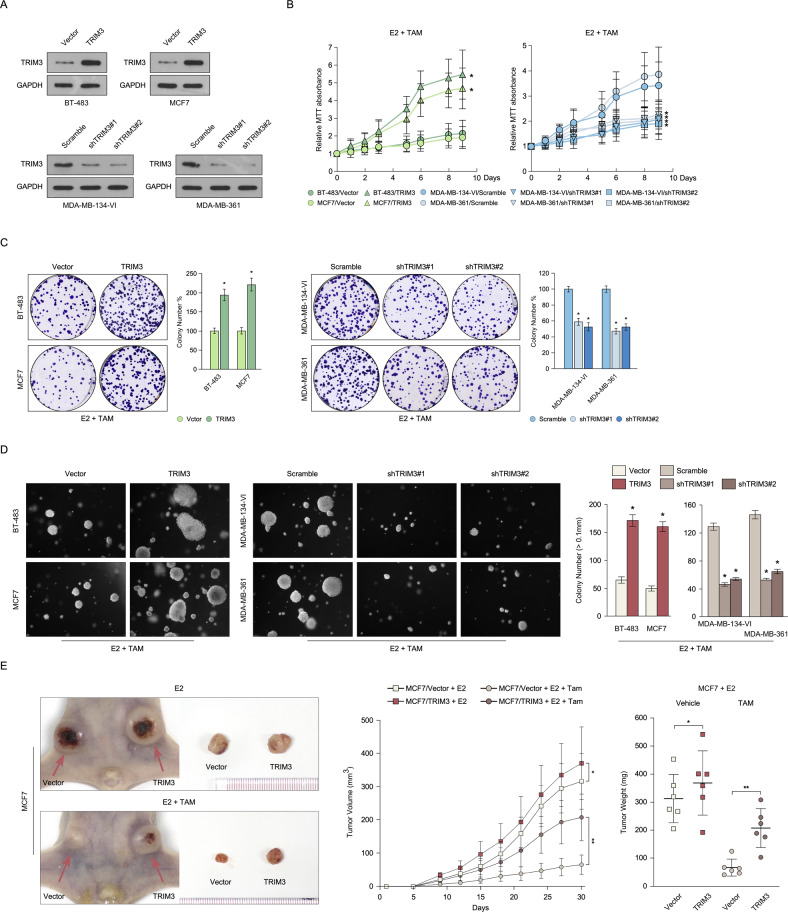
TRIM3 contributes to tamoxifen resistance in breast cancer. **A** Western blotting analysis of TRIM3 levels in the indicated breast cancer cell lines stably overexpressing TRIM3 and silencing for TRIM3. Cell viability was measured using MTT (**B**) and colony formation (**C**) assays in the indicated cell lines treated with E2 (10 nM) and TAM (1 μM). Statistical data are presented as the mean ± SD. **D** Soft agar assay of the indicated cells treated with E2 (10 nM) and TAM (1 μM) (left panel) and the statistical significance of colony formation was assessed in the right panel. Data are presented as the mean ± SD. *P* < 0.05. **E** Representative images of the tumors in the xenografts (left panel). Tumor growth curves (middle panel) and tumor weight (right panel) of the indicated xenograft tumors (*n* = 6/group). Data are presented as the mean ± SD. *P < 0.05, **P < 0.01.

To further determine the role of TRIM3 in regulating tamoxifen resistance, we detected the expression levels of TRIM3 in 12 ER^+^ breast cancer cell lines. As shown in Fig. S2A, TRIM3 was highly expressed in MDA-MB-134-VI, MDA-MB-175-VII, and MDA-MB-361 cells, and showed low expression in BT-483, CAMA-1, and MCF7 cells. Consistent with the clinical evidence, higher expression of TRIM3 in MDA-MB-134-VI, MDA-MB-175-VII, and MDA-MB-361 cells was associated with resistance to tamoxifen compared with lower expression of TRIM3 in BT-483, CAMA-1, and MCF7 (Fig. S2B, C), suggesting that TRIM3 might contribute to tamoxifen resistance in breast cancer. Furthermore, we established BT-483 and MCF7 cells that stably overexpressed TRIM3, and MDA-MB-134-VI and MDA-MB-361 that were stably silenced for TRIM3 (Fig. 2A). As expected, overexpression of TRIM3-induced tamoxifen resistance and resulted in more colony formation in BT-483 and MCF7 cells, whereas silencing TRIM3 expression promoted tamoxifen sensitivity and resulted in less colony formation in MDA-MB-134-VI and MDA-MB-361 cells (Fig. 2B, C). Moreover, endogenous TRIM3 expression was upregulated in tamoxifen-resistance MCF7 cells (MCF7/TR), and silencing its expression reversed tamoxifen resistance (Fig. S3A–C). Therefore, these results revealed that TRIM3-induced tamoxifen resistance in ER^+^ breast cancer in vitro.

### TRIM3 confers tamoxifen resistance in ER^+^ breast cancer in vivo

To further investigate the function of TRIM3 in regulating tamoxifen resistance in vivo, we first examined the anchor-independent growth ability by manipulation of TRIM3 expression in cells compared with control cells. As shown in Fig. 2D, TRIM3 overexpression promoted cell growth and the cells formed more and larger colonies compared with vector-transformed cells, whereas silencing TRIM3 expression inhibited cell growth and the cells formed fewer and smaller colonies compared with vector-transformed cells. Furthermore, we inoculated TRIM3-deregulated cells subcutaneously into athymic nude mice. Consistently, TRIM3 overexpressing cells formed larger tumors compared with control vector-transformed cells (Fig. 2E). Taken together, these results revealed that TRIM3 responds to tamoxifen resistance and promotes tumorigenicity of breast cancer in vivo.

### TRIM3 promotes SUMOylation of ESR1 via binding to UBC9

**Fig. 3:**
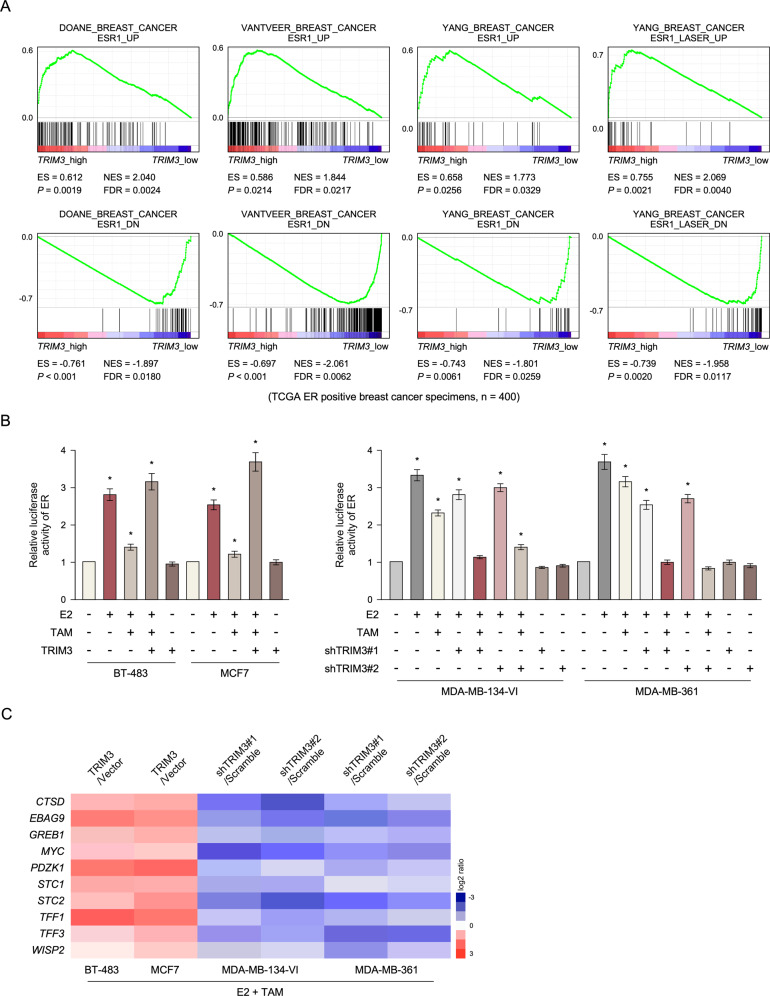
TRIM3-induced estrogen receptor (ER)-dependent transcription in response to tamoxifen therapy. **A** GSEA showed that *TRIM3* expression correlated positively with ESR1-activated gene signatures and negatively with ESR1-suppressed gene signatures in ER^+^ breast cancer profiles (TCGA, *n* = 400). **B** Estrogen response element (ERE) luciferase reporter activity was analyzed in the indicated cells treated with E2 (10 nM) and/or TAM (1 μM). Data are presented as the mean ± SD. *P < 0.05. **C** Heatmap of ER-regulated gene expression in the indicated cells treated with E2 and TAM.

To further investigate the mechanism by which TRIM3 responds to tamoxifen resistance, gene set enrichment analysis was analyzed in published ER^+^ breast cancer expression profiles from TCGA data. We found that *TRIM3* levels correlated positively with *ESR1* expression, suggesting that TRIM3 might be involved in regulating the ESR signaling pathway (Fig. 3A). As expected, we found that overexpression of TRIM3 increased estrogen response element (ERE)-driven luciferase reporter activity and promoted ER-regulated gene expression upon tamoxifen treatment, whereas silencing of TRIM3 decreased their expression (Fig. 3B, C). However, analysis of breast cancer data in the TCGA showed that levels of TRIM3 mRNA and protein did not correlate with levels of ESR1 mRNA and protein (Fig. S4A, S5A). Furthermore, we found that E2 treatment did not induce ESR1 and TRIM3 expression in breast cancer (Fig. S4B). Overexpression of ESR1 did not affect the expression of TRIM3 (Fig. S4C). Collectively, TRIM3-induced ESR1 transcriptional activity, but did not induce ESR1 expression.

**Fig. 4:**
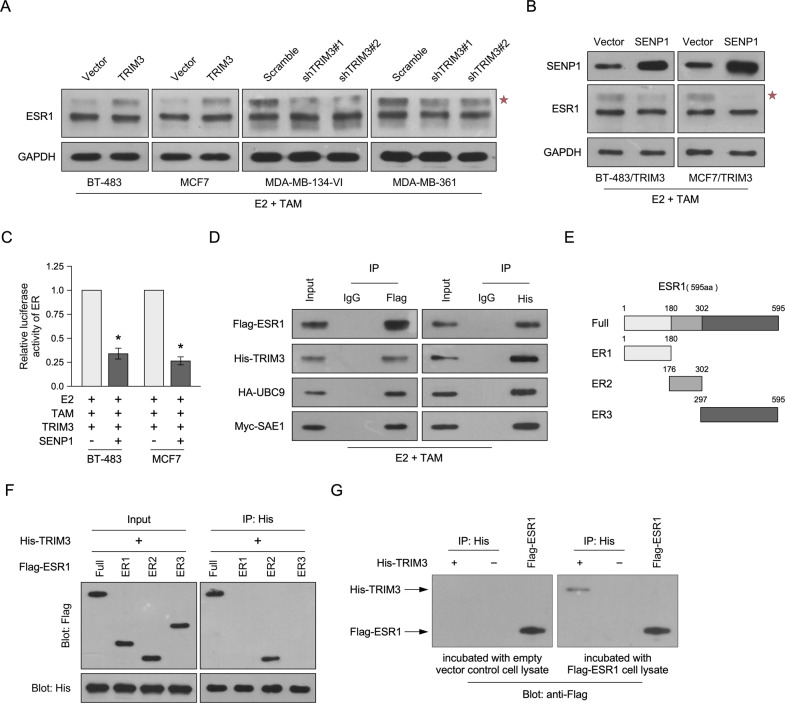
TRIM3-induced SUMOylation of ESR1 via binding with UBC9. Western blotting of ESR1 (**A**, **B**) and SENP1 (**B**) levels in the indicated breast cancer cell lines. GAPDH was used as the loading control. A star represents SUMOylation of ESR1. **C** Estrogen response element (ERE) luciferase reporter activity was analyzed in the indicated cells treated with E2 (10 nM) and/or TAM (1 μM). Data are presented as the mean ± SD. *P < 0.05. **D** Immunoprecipitation assay revealing that TRIM3 interacts with ESR1, UBC9, and SAE1 in the indicated cells. **E** Schematic illustration of wild-type ESR1 and truncated ESR1. **F** Immunoprecipitation assay revealing that TRIM3 interacts with the ER2 domain of ESR1. **G** Far-western blotting revealing that TRIM3 directly interacts with Flag-ESR1 in the cell lysate.

The E3 ubiquitin-protein ligase TRIM proteins are a new class of SUMO ligases (E3s), which regulate SUMOylation specificity [18]. The transcription activity of ERα is upregulated by SUMOylation via binding to UBC9, the unique SUMO E2-conjugating enzyme, and is repressed by tamoxifen treatment [16, 22–24]. Here, we found that TRIM3 overexpression increased SUMO modification of ESR1, whereas silencing TRIM3 decreased SUMOylation (Fig. 4A). Overexpression of SENP1, a deSUMOylase enzyme, decreased the SUMOylation of ESR1 and ERE luciferase reporter activity in TRIM3-transduced cells compared with control cells upon tamoxifen treatment (Fig. 4B, C). Furthermore, immunoprecipitation and western blotting assays showed that TRIM3 formed a complex with ESR1 and UBC9, suggesting that TRIM3 might regulate the SUMOylation of ESR1 by binding to ESR1, UBC9, and SUMO1 activating enzyme subunit 1 (SAE1) (Fig. 4D). Silencing UBC9 in TRIM3-transduced cells reduced the SUMOylation of ESR1 and ERE luciferase reporter activity (Fig. S5B, C). To further investigate the binding of TRIM3 and ESR1, three truncated ESR1 fragments were constructed (Fig. 4E). Immunoprecipitation and far-western blotting assays showed that TRIM3 interacted with the DNA-binding fragment of ESR1 (ER2 fragment) and directly interacted with Flag-tagged ESR1 cell lysate (Fig. 4F, G). Taken together, these data revealed that TRIM3 promotes SUMOylation and activates the transcription activity of ESR1 by interacting with ESR1 and UBC9.

### UBC9 is required for tamoxifen resistance effect of TRIM3

**Fig. 5:**
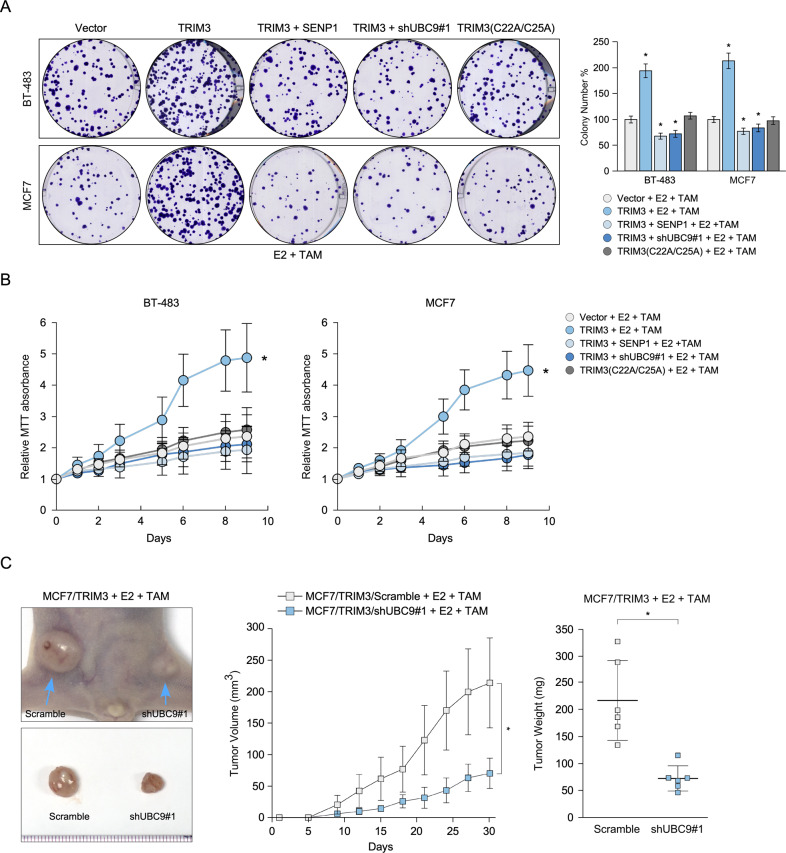
Silencing UBC9 reverses the tamoxifen resistance effect of TRIM3. Representative images of colony formation (**A**) and MTT assays (**B**) of the indicated cells treated with E2 (10 nM) and TAM (1 μM). Histograms showing colonies formed by the indicated cells. Data are presented as the mean ± SD. *P < 0.05. **C** Representative images of the tumors in the xenografts (left panel), tumor growth curves (middle panel) and tumor weight (right panel) of the indicated xenograft tumors (*n* = 6/group). Data are presented as the mean ± SD. *P < 0.05.

To further investigate whether SUMOylation of ESR1 is required to promote the tamoxifen resistance effect of TRIM3, the effect of silencing of UBC9 and overexpressing SENP1 on TRIM3-induced tamoxifen resistance were examined. As shown in Fig. 5A, B, silencing UBC9 and overexpressing SENP1 in TRIM3-transduced cells resulted in formed reduced colony formation, which promoted tamoxifen sensitivity. Furthermore, silencing UBC9 in TRIM3-transduced cells reversed the ability of TRIM3 to induce tamoxifen resistance and decreased tumorigenicity in vivo (Fig. 5C). Consistently, the catalytic dead mutant TRIM3 (C22A/C25A) rescued tamoxifen sensitivity in vitro and in vivo, implying that regulation of TRIM3 in tamoxifen resistance was dependent on its catalytic activity (Fig. 5A, B, Fig. S6). Taken together, these results suggest that UBC9 is required for TRIM3 regulation of ESR1 SUMOylation and the promotion of tamoxifen resistance in breast cancer.

### TRIM3 correlates with ESR1 SUMOylation and tamoxifen resistance in breast cancer

**Fig. 6:**
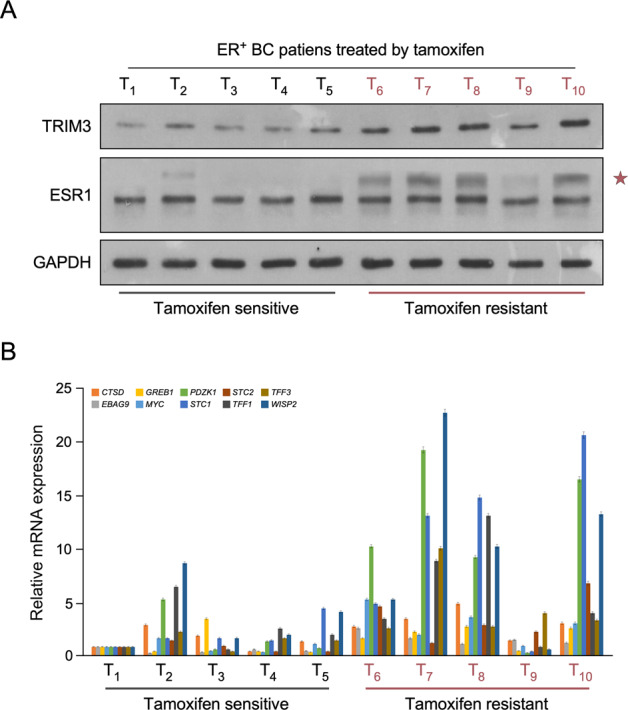
TRIM3 promotes transcription of ER-target genes in clinical breast cancer. Western blotting of TRIM3 and ESR1 (**A**), qPCR of ER-targeted genes (**B**) in 10 cases of freshly ER^+^ breast cancer treated with tamoxifen therapy, including tamoxifen-resistant and tamoxifen-sensitive tissues. GAPDH was used as the loading control. A star represents SUMOylation of ESR1.

To further investigate the correlation of TRIM3 and ESR1 in regulating tamoxifen resistance, 10 freshly collected clinical breast cancer samples were examined. As shown in Fig. 6A, TRIM3 expression was upregulated in tamoxifen-resistant breast cancer compared with that in tamoxifen-sensitive breast cancer, and was strongly associated with ESR1 SUMOylation. Consistently, TRIM3 promoted the transcription of ER-targeted genes in tamoxifen-resistant ER^+^ breast cancer (Fig. 6B). Collectively, these results support the view that TRIM3 upregulation promotes ESR1 SUMO modification and ER signaling pathway activity, leading to tamoxifen resistance in ER^+^ breast cancer.

## Discussion

Tamoxifen is a widely used treatment for patients with ER-positive breast cancer; however, de novo or acquired resistance results in failure of tamoxifen therapy, which is a clinical challenge [4, 5]. In the present study, we found that TRIM3 was upregulated in tamoxifen-resistant breast cancer tissues compared with that tamoxifen-sensitive breast cancer tissues, and correlated closely with poorer survival in ER^+^ breast cancer. Overexpression of TRIM3 contributed to cell survival upon tamoxifen treatment both in vivo and in vitro. Moreover, TRIM3 promoted the ESR1 SUMO modification and activated the transcription of ER-target genes by binding to UBC9, which could be abolished by overexpression of SENP1. Taken together, our results demonstrated that TRIM3 modifies the SUMOylation of ESR1 and confers tamoxifen resistance, which provided a novel therapeutic target for breast cancer therapy.

TRIM3, a member of RING-type E3 ubiquitin ligases, plays diverse roles in regulating neoplastic processes [21, 25–27]. TRIM3 was identified as a novel RING finger protein expressed in the rat brain, which is involved in regulating neuronal outgrowth [19, 28]. TRIM3 is a candidate brain tumor suppressor gene, which suppresses brain tumorigenesis via attenuating Notch signaling and suppressing *c-MYC* expression [29, 30]. Furthermore, TRIM3 is downregulated in various types of cancer, including liver cancer [20], esophageal squamous cell carcinoma [27], and colon cancer [31], and inhibits cell proliferation, invasion, and metastasis. However, the correlation between TRIM3 and tamoxifen resistance in breast cancer remains unclear. In the current study, we found that TRIM3 showed significantly higher expression in recurrent ER^+^ breast cancer than that in non-recurrent ER^+^ breast cancer, which consistent with the analysis of TRIM3 expression in response to tamoxifen treatment in ER^+^ breast cancer data obtained from the TCGA (Figs. 1A, B and 6A). Furthermore, the expression of *TRIM3* correlated with poorer OS, RFS, and DMFS in patients with ER^+^ breast cancer upon tamoxifen treatment, but not in patients with ER^−^ breast cancer (Figs. 1C and S1C), suggesting that TRIM3 might be a potential predictive marker for the response to tamoxifen in patients with ER^+^ breast cancer.

ERα has been recognized as a favorable prognostic biomarker and has a determinative role for breast cancer therapy [32]. ERα belongs to the nuclear receptor superfamily, and activates the transcription of targeted genes to resist tamoxifen treatment [33, 34]. SUMOylation, as a PTM, is hyperactivated in breast cancer and correlates with ERα signaling pathways [35]. Although SUMO modification represses transcription via binding with co-repressors, including HDACs and DAXX [36, 37], growing evidence has shown that SUMOylation is involved in promoting transcriptional activity. For example, the transcriptional activity of ERα is upregulated in breast cancer via binding with SUMOylation proteins, such as CLOCK [22] and ZFP282 [38]. Furthermore, ERα was proven to be SUMOylated via binding with UBC9, and blocking its SUMOylation impaired the transcriptional activity of ERα through decreased DNA binding, without influencing the cellular localization of ERα [16]. Therefore, the precise molecular mechanism by which SUMO modification enhances the transcriptional activity of ERα in breast cancer requires further investigation.

In the present study, we identified that TRIM3 plays an important role in regulating the ER signaling pathway and confers tamoxifen resistance in breast cancer. Overexpression of TRIM3 enhanced ERE luciferase reporter activity and upregulated ER-regulated genes upon tamoxifen treatment, whereas silencing of TRIM3 had the opposite effect (Fig. 3B, C). Furthermore, TRIM proteins regulate SUMO modification by transferring SUMO1 from UBC9 to the substrate [17, 18]. Consistently, the SUMOylation of ESR1 was upregulated in TRIM3-transduced cells upon tamoxifen treatment, which was abolished by overexpression of SENP1 (Fig. 4A, C). Furthermore, immunoprecipitation analysis demonstrated that TRIM3 interacted with ESR1, UBC9, and SAE1, suggesting that TRIM3 acts as a SUMO E3 ligase to transfer SUMO from SAE1 (E1) and UBC9 (E2) to ESR1 (substrate) (Fig. 4D). Importantly, TRIM3 interacted with ESR1 by binding directly to its DNA-binding domain (Fig. 4E–G), which might induce ESR1 transcriptional activity. Overexpression of UBC9 promoted breast cancer tumorigenesis, while a dominant negative mutant of UBC9 decreased tumorigenesis [39]. Similarly, blocking UBC9 expression also decreased cell survival and tumorigenesis in TRIM3-transduced cells upon E2 and tamoxifen treatment in vitro and in vivo (Fig. 5). To further examine the effect of catalytic dead mutant of TRIM3 in tamoxifen response in ER^+^ breast cancer, we constructed TRIM3 mutant (C22A/C25A), which affects its E3 ligase activity [2, 3]. Consistently, TRIM3 (C22A/C25A) rescued tamoxifen sensitivity in vitro and in vivo (Fig. 5A, B, Fig. S6). Therefore, these results suggest that regulation of TRIM3 tamoxifen resistance was dependent on its catalytic activity.

In conclusion, our results confirmed that TRIM3 acts as a SUMO E3 ligase to regulate ESR1 SUMO modification and transcriptional activity, thus conferring tamoxifen resistance via the TRIM3/UBC9/ESR1 axis in ER^+^ breast cancer. Silencing UBC9 in TRIM3-transduced cells conferred tamoxifen sensitivity in breast cancer. Importantly, TRIM3 overexpression correlated with poor survival of patients with ER^+^ breast cancer with tamoxifen resistance, identifying TRIM3 as a potential biomarker for the treatment of ER^+^ breast cancer.

## Materials and methods

### Cell culture

The human breast cancer cell lines BT-474, BT-483, CAMA-1, HCC1428, HCC1500, MCF7, MDA-MB-134-VI, MDA-MB-175-VII, MDA-MB-361, T-47D, ZR-75-1 ZR-75-30, and MCF/TR were cultured according to the manufacturer’s instruction. Short tandem repeat (STR) profiling were used and authenticated in all cell lines. Mycoplasma eradication was evaluated by PCR.

### Chemical reagents

17β-estradiol (E2) and 4-hydroxytamoxifen (TAM) were purchased from Sigma-Aldrich (St. Louis, MO, USA).

### Tissue specimens and immunohistochemistry

A cohort of paraffin-embedded breast cancer (48 cases) which received tamoxifen therapy was used to detect the expression of TRIM3 via Anti-TRIM3 (ab111840). The specimens were obtained from the First Affiliated Hospital of Sun Yat-sen University between 2010 and 2015. 10 freshly breast cancer tissues with tamoxifen-sensitive and tamoxifen resistant were collected. Prior patient consent and approval were obtained.

### Constructs and transfection

Genomic DNA of TRIM3 was PCR-amplified and cloned into a pBABE-puro retroviral vector. TRIM3 (C22A/C25A) was generated and cloned into a pBABE-puro retroviral vector. The cloned into a pBABE-puro retroviral vector.e pSUPER-puro shRNA of TRIM3 and UBC9 were purchased from Transheep Bio. All clone primer and siRNA oligonucleotides are listed as Table S2. Estrogen Response Element (ERE) was cloned into a pGL3 basic vector (Promega). The Renilla luciferase TK was used as transfection control. All cells overexpressing TRIM3 or silencing TRIM3 were selected with 0.5 μg/ml puromycin.

### Immunoprecipitation analysis

Immunoprecipitation assay was performed according to described previously [40]. Lysates were incubated with Flag or His affinity beads (Sigma-Aldrich). The agarose beads were washed with wash buffer. Then the eluations were detected using appropriate antibodies.

### Far-western blotting

Far immunoblotting was performed by using the proteins immunoprecipitated by anti-His antibody. The proteins were detected using western blotting. Empty vector or Flag-ESR1 cell lysate was added to the PVDF membrane and incubated overnight. Then the membrane was subjected to immunoblotting analysis by indicated antibody.

### Western blotting analysis

Western blotting was performed using antibodies against TRIM3 (ab111840), ESR1 (ab108398), UBC9 (ab75854), SENP1 (ab108981) and SAE1 (ab185552). The membranes were re-probed with an anti-GAPDH (BOSTER, BM3876) as the loading control.

### Quantitative real-time reverse transcription PCR (qPCR)

Total RNA was isolated with TRIzol reagent (Invitrogen) according to manufacturer’s instructions. RNA was reverse-transcribed into cDNA and carried out via Real-time PCR with SYBR Green Master (Roche). The data were assessed base on the threshold cycle (Ct), and calculated as 2^−[(*C*^_t_^of gene) – (*C*^_t_^of *GAPDH*)]^, which was normalized to GAPDH expression. All primers are listed as Table S2.

### Cell viability assay

Indicated cells were treated with Estrogen and/or tamoxifen for 48 h. The cells were added MTT dye (Sigma-Aldrich), and then the cells were resuspended with dimethyl sulfoxide (Sigma-Aldrich) and measured with automatic microplate reader.

### Colony formation assay

Indicated cells were incubated at a level of 5% CO_2_ at a temperature of 37 °C for 2 weeks. Then the cells were fixed, stained with crystal violet stain, and counted.

### Luciferase activity assay

Luciferase reporter plasmid and pRL-TK Renilla plasmid were transfected into indicated cells. After 48 h, the cells were lysis and measured using a Dual Luciferase Reporter Assay (Promega) according to the manufacturer’s instructions.

### Anchorage-independent growth ability assay

Indicated cells were suspended with medium plus 0.33% agar, and then plated on top of 0.66% agar medium mix. After 10 days, colonies >0.1 mm in diameter were counted. The experiments were performed in triplicates.

### Tumor xenografts

Under the guideline of National Institutes of Health Guide for Care and Use of Laboratory Animals, xenografts were performed using athymic nude female mice (4–5 weeks of age, 18–20 g). 5 × 10^6^ of indicated cells were injected into the left and right dorsal flank of mice implanted with E2 pellets (0.72 mg/pellet; 60-day release). After 1 week, a subcutaneous injection with or without tamoxifen pellet (5 mg/pellet; 60-day release). Tumor were examined twice a week, and tumor volume was calculated as (L × W^2^)/2.

### Data processing and visualization

The dataset is available in TCGA (https://tcga-data.nci.nih.gov/tcga/). Gene set enrichment analysis (GSEA) was performed on GSEA 2.0. 9 (http://www.broadinstitute.org/gsea/). The relationship between the expression of TRIM3 and ESR1 was determined by correlation coefficient. For the relationship between *TRIM3* and the OS, RFS and DMFS of breast cancers, Kaplan–Meier Plotter (http://kmplot.com/analysis) was used.

### Statistics’

Unpaired Student’s test was used to evaluate the statistical significance of the differences between two groups. Spearman’s correlation analysis and Chi-square were used to evaluate the correlations. All *P* values were two-tailed, and a value of *P* < 0.05 was considered statistically significant. All data analyze were performed with SPSS19.0 software and presented with GraphPad Prism 8.0.

## Supplementary information

Supplemental

Supplemental Fig. S1

Supplementary Fig. S2

Supplementary Fig. S3

Supplemental Fig. S4

Supplemental Fig. S5

Supplementary Fig. S6
