# Supplementary material for: Tripartite motif-containing 3 (TRIM3) enhances ER signaling and confers tamoxifen resistance in breast cancer
Source: Oncogenesis. 2021 Sep 10;10(9):60. doi: 10.1038/s41389-021-00350-x (PMC8433133; doi:10.1038/s41389-021-00350-x)
Supplement: Supplementary file 1 — Supplemental [file 41389_2021_350_MOESM1_ESM.docx]

**Supplemental Information**

**Supplemental Table 1. Clinicopathological characteristics and expression of TRIM3 in breast cancer patients**

|  | **Number of cases** |
| --- | --- |
| **Age(years)** |  |
| < 55 | 27 |
| ≥ 55 | 21 |
| **Menopause** |  |
| Yes | 22 |
| Not | 26 |
| **Clinical Stage** |  |
| I | 18 |
| IIa | 15 |
| IIb | 9 |
| IIIa | 3 |
| IIIb | 1 |
| IV | 2 |
| **T classification** |  |
| T1 | 24 |
| T2 | 20 |
| T3 | 3 |
| T4 | 1 |
| **N classification** |  |
| N0 | 28 |
| N1 | 16 |
| N2 | 1 |
| N3 | 1 |
| N4 | 2 |
| **M classification** |  |
| Yes | 2 |
| Not | 46 |
| **Molecular classification** |  |
| Luminal A | 39 |
| Luminal B | 9 |
| **Recurrence**  Yes  Not | 14  34 |
| **Survive or Mortality** |  |
| Survive | 35 |
| Mortality | 13 |

**Supplemental Table 2: Primers and Oligonucleotides**

| **Used for subcloning and plasmid construction:** | |
| --- | --- |
| pSUPER retro-puro-sh-TRIM3-F1: | GATCCCCGCTCACTGTCACTACCAAATTCAAGAGATTTGGTAGTGACAGTGAGCTTTTTA |
| pSUPER retro-puro-sh-TRIM3-R1: | AGCTTAAAAAGCTCACTGTCACTACCAAATCTCTTGAATTTGGTAGTGACAGTGAGC GGG |
| pSUPER retro-puro-sh-TRIM3-F2: | GATCCCCCCACAAGAATGGCACATATTTCAAGAGAATATGTGCCATTCTTGTGGTTTTTA |
| pSUPER retro-puro-sh-TRIM3-R2: | AGCTTAAAAACCACAAGAATGGCACATATTCTCTTGAAATATGTGCCATTCTTGTGGGGG |
| pBABE-puro-TRIM3-F(BamHI) | ctctaggcgccggccggatccATGGCAAAGAGGGAGGACAGC |
| pBABE-puro-TRIM3-R(SnaBI) | gtgctggcgaattcctacgtaCTACTGGAGGTAGCGATAGGCTTT |
| UBC9 shRNA clone primer up | GATCCCCCCATCTTAGAGGAGGACAATTCAAGAGATTGTCCTCCTCTAAGATGGTTTTTA |
| UBC9 shRNA clone primer dn | AGCTTAAAAACCATCTTAGAGGAGGACAATCTCTTGAATTGTCCTCCTCTAAGATGGGGG |
| **Used for qPCR** | |
| TRIM3 real time primer up | GCGACCTGGAGACCATTTGT |
| TRIM3 real time primer dn | GCTACTGCCGATGTGTTCCTG |
| EBAG9 real time primer up | AGTTCCTAAGCAGACAGATGTTG |
| EBAG9 real time primer dn | CCCATTCCCTCCTTCGATCTTTA |
| GREB1 real time primer up | ATGGGAAATTCTTACGCTGGAC |
| GREB1 real time primer dn | CACTCGGCTACCACCTTCT |
| MYC real time primer up | GGCTCCTGGCAAAAGGTCA |
| MYC real time primer dn | CTGCGTAGTTGTGCTGATGT |
| PDZK1 real time primer up | TTCCTGCGAATTGAGAAGGAC |
| PDZK1 real time primer dn | TCCACCCGTGTTTTCACTGC |
| STC1 real time primer up | GTGGCGGCTCAAAACTCAG |
| STC1 real time primer dn | GTGGAGCACCTCCGAATGG |
| STC2 real time primer up | GGGTGTGGCGTGTTTGAATG |
| STC2 real time primer dn | TTTCCAGCGTTGTGCAGAAAA |
| TFF1 real time primer up | CCCCGTGAAAGACAGAATTGT |
| TFF1 real time primer dn | GGTGTCGTCGAAACAGCAG |
| TFF3 real time primer up | CCAAGCAAACAATCCAGAGCA |
| TFF3 real time primer dn | GCTCAGGACTCGCTTCATGG |
| GAPDH real time primer up | GGAGCGAGATCCCTCCAAAAT |
| GAPDH real time primer dn | GGCTGTTGTCATACTTCTCATGG |

**Supplemental Figure Legends**

**Supplemental Fig. S1. TRIM3 correlates with poor survival in breast cancer profile from TCGA**. (A) A boxplot representing *TRIM3* expression level in ER^+^ breast cancer higher than ER^-^ breast cancer and normal breast tissue. Data are presented as mean ± SD. (B) A plot showing the relation between TRIM3 mRNA, protein and CNV in breast cancer profile from TCGA. Spearman correlation :0.66 (*P*=7.69e-8). Person:0.74 (*P*=4.62e-10). (C) Kaplan-Meier (KM) plotter analysis of correlation of *TRIM3* expression with OS, RFS and DMFS in ER^+^ breast cancer with tamoxifen and chemotherapy treatment and ER^-^ breast cancer.

**Supplementary Fig. S2. The effect of TRIM3 on tamoxifen response in breast cancer.** (A) mRNA and protein levels of TRIM3 in ER^+^ breast cancer cell lines. Data are presented as the mean ± SD. (B-C) Cell viability was measured using an MTT (B) and colony formation (C) assays in the indicated cell lines treated with E2 (10 nM) and/or TAM (1 μM). Statistical data are presented as the mean ± SD. **P* < 0.05.

**Supplementary Fig. S3. In vitro effects of TRIM3 on tamoxifen response in breast cancer.** (A) Western blotting analysis of TRIM3 levels in the indicated breast cancer cell lines. (B-C) Cell viability was measured using MTT (B) and colony formation (C upper panel) and Soft agar (C lower panel ) assays in the indicated cell lines treated with E2 (10 nM) and TAM (1 μM). Statistical data are presented as the mean ± SD. **P* < 0.05.

**Supplemental Fig. S4. Correlation of expression of TRIM3 and ESR1 in data base and in indicated cells.** (A) A plot showing no relation between *TRIM3* mRNA levels and ESR1 mRNA and protein levels in breast cancer profile from TCGA. (B-C) Western blotting assay showing the expression of ESR1 and TRIM3 in indicated cells treated with E2 (10 nM).

**Supplemental Fig. S5. Silencing UBC9 decreased activity of ESR1.** (A) A plot showing no relation between TRIM3 protein levels and ESR1 protein levels in breast cancer profile from TCGA. (B) Western blot showing expression of UBC9, TRIM3 and ESR1 in indicated cells treated with E2 (10 nM) and TAM (1μM). GAPDH was used as loading control. A star represents SUMOylation of ESR1. (C) Estrogen response element (ERE) luciferase reporter activity was analyzed in indicated cells treated with E2 (10 nM) and TAM (1μM). Data are presented as mean ± SD. **P*<0.05.

**Supplementary Fig. S6. In vivo effects of catalytically inactive TRIM3 (C22A/C25A) on tamoxifen response in breast cancer.** Representative images of the tumors in the xenografts (left panel), tumor growth curves (middle panel) and tumor weight (right panel) of the indicated xenograft tumors (n = 6 /group). Data are presented as the mean ± SD. **P* < 0.05.
