# Supplementary figures and images for: Tripartite motif-containing 3 (TRIM3) enhances ER signaling and confers tamoxifen resistance in breast cancer
Source: Oncogenesis. 2021 Sep 10;10(9):60. doi: 10.1038/s41389-021-00350-x (PMC8433133; doi:10.1038/s41389-021-00350-x)

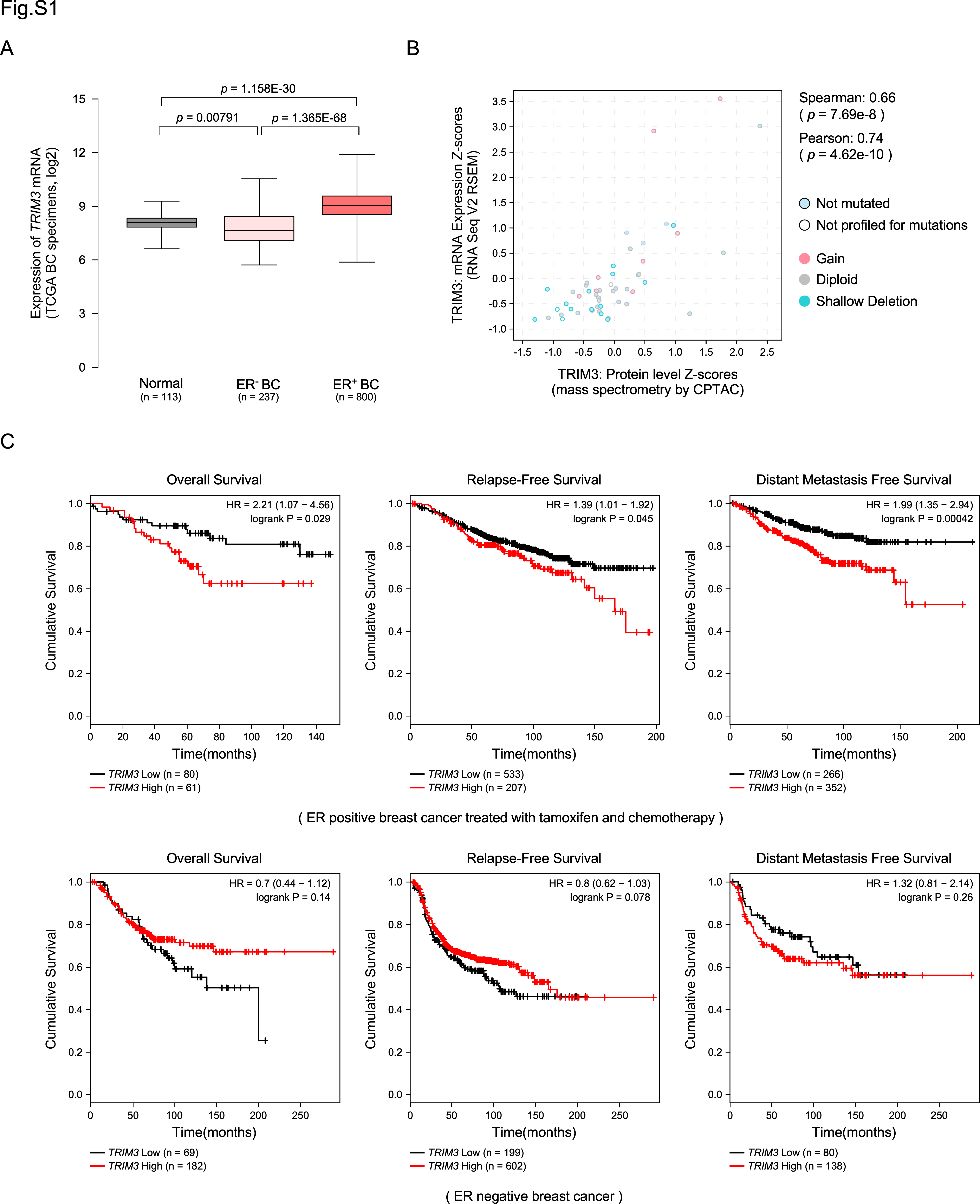

Supplement: Supplementary file 2 — Supplemental Fig. S1 [file 41389_2021_350_MOESM2_ESM.tif]

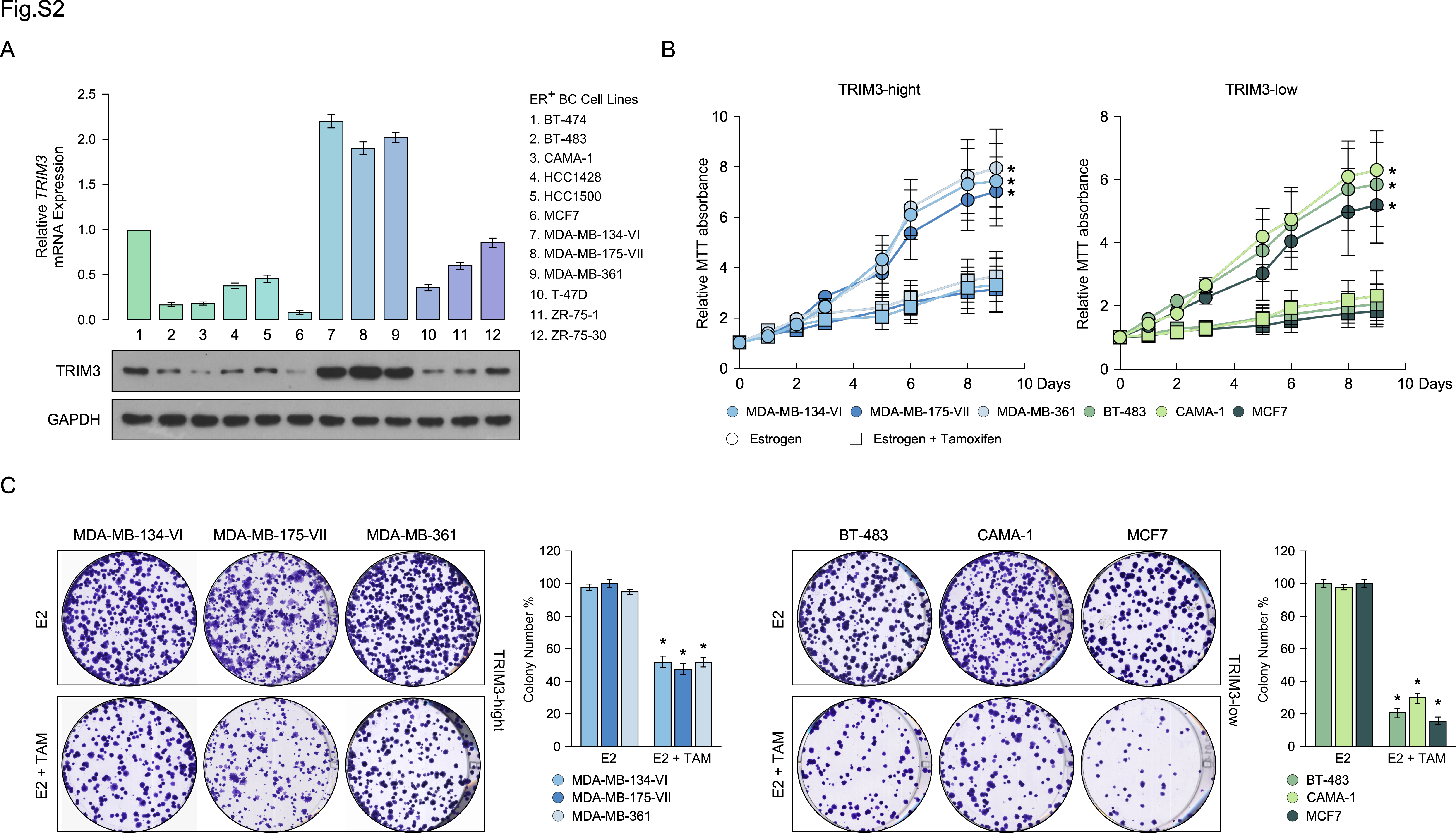

Supplement: Supplementary file 3 — Supplementary Fig. S2 [file 41389_2021_350_MOESM3_ESM.tif]

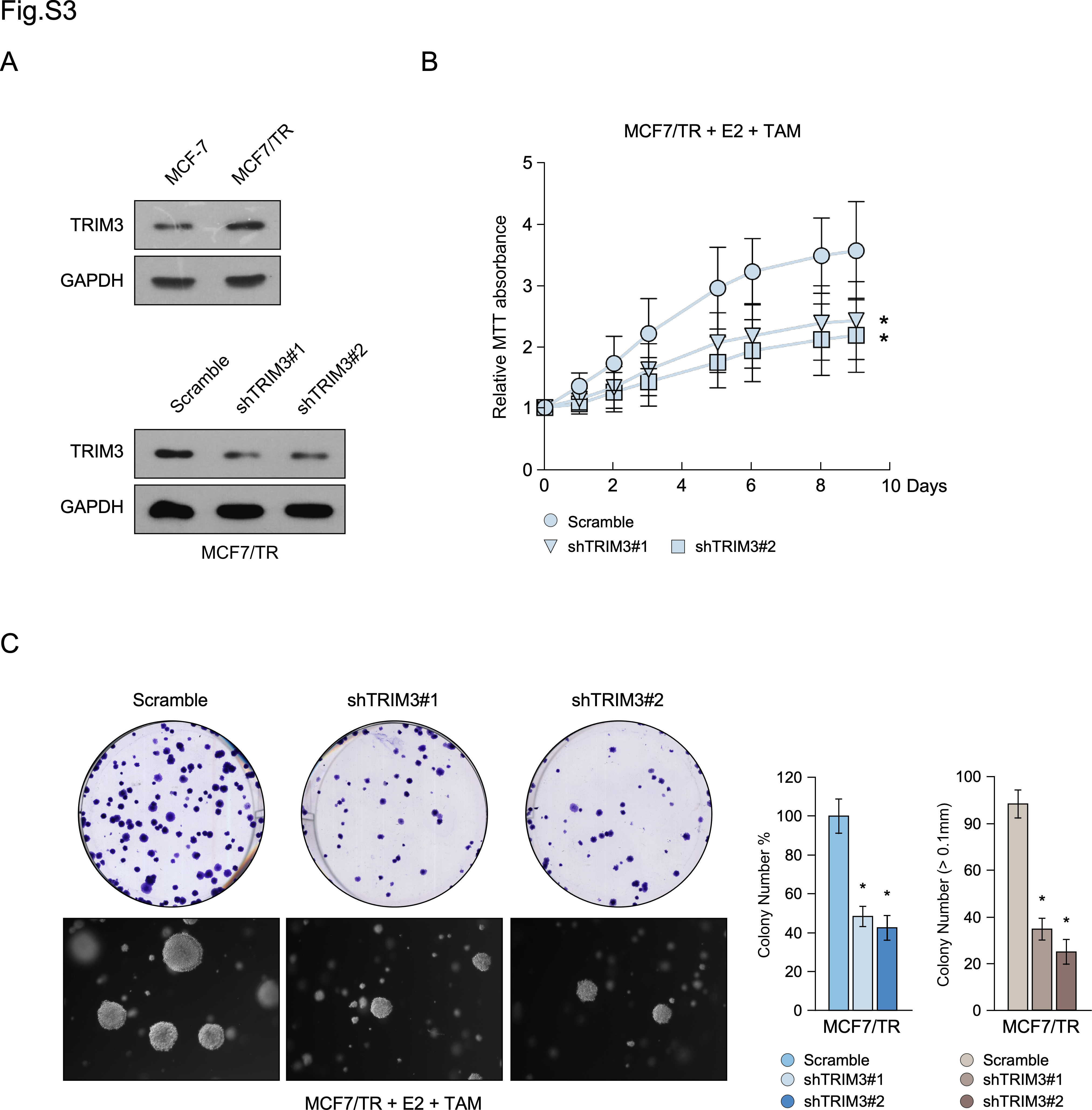

Supplement: Supplementary file 4 — Supplementary Fig. S3 [file 41389_2021_350_MOESM4_ESM.tif]

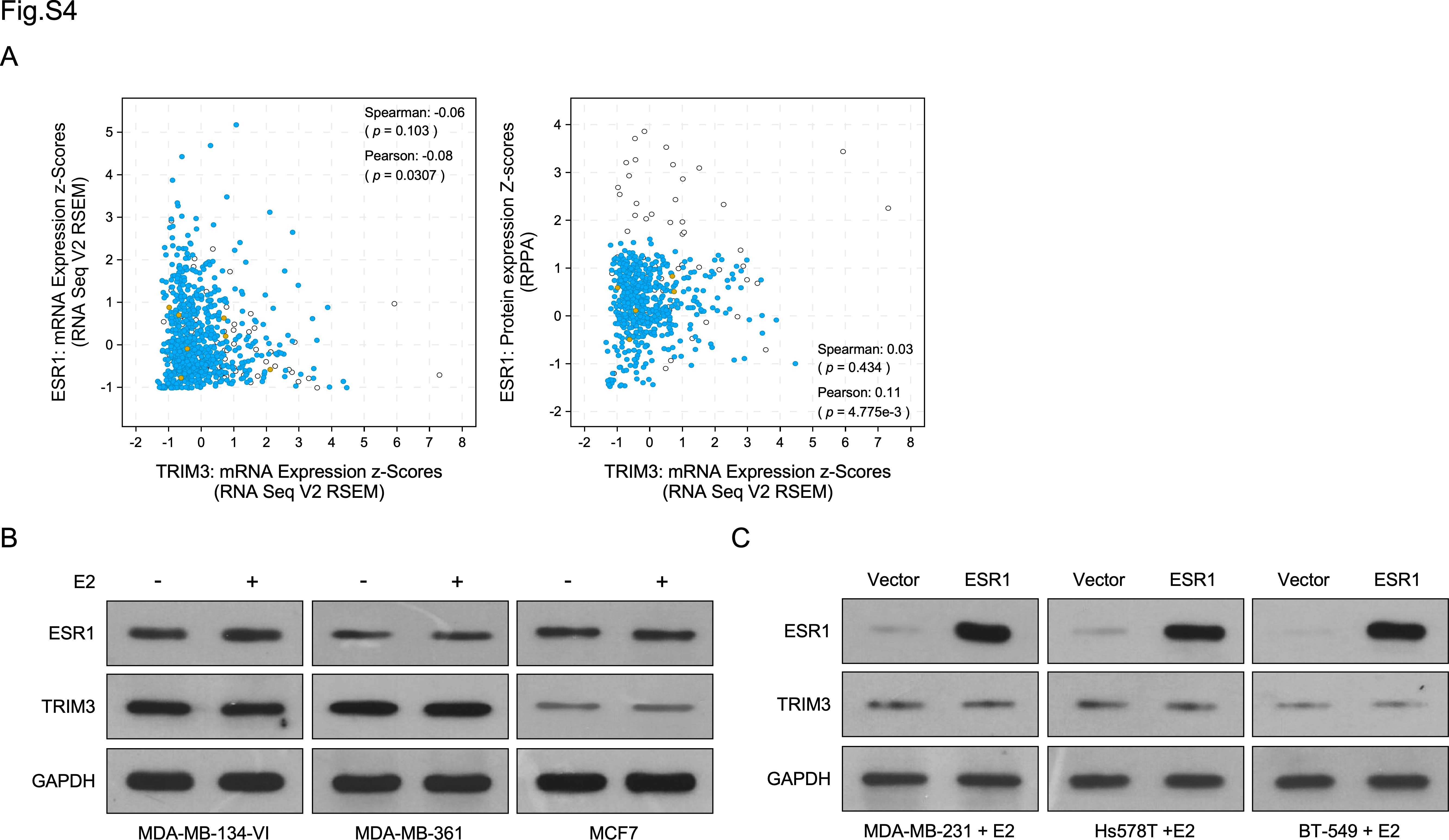

Supplement: Supplementary file 5 — Supplemental Fig. S4 [file 41389_2021_350_MOESM5_ESM.tif]

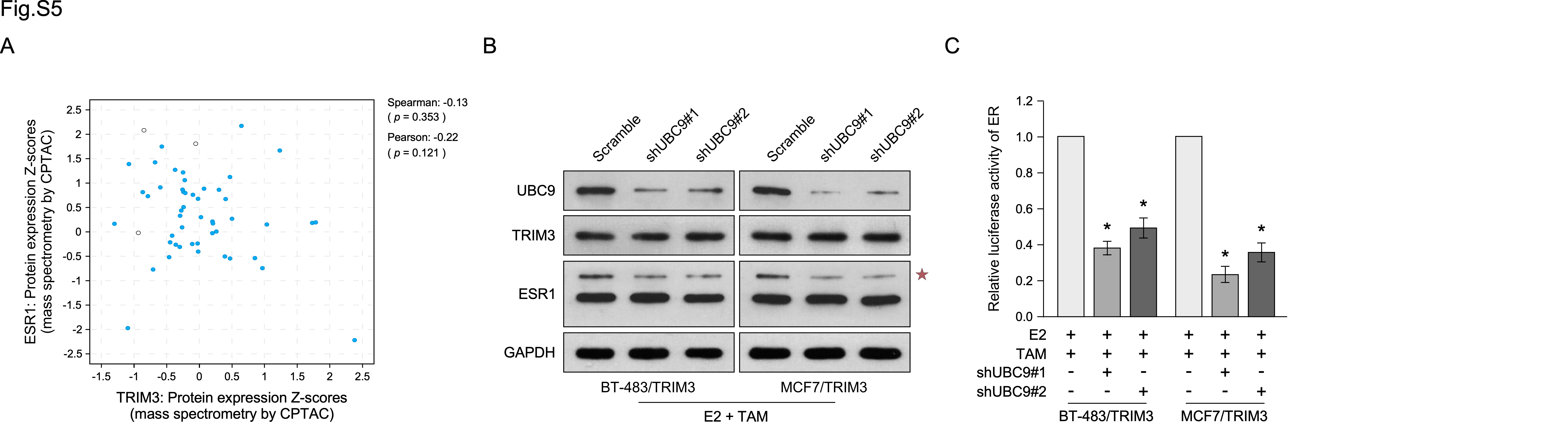

Supplement: Supplementary file 6 — Supplemental Fig. S5 [file 41389_2021_350_MOESM6_ESM.tif]

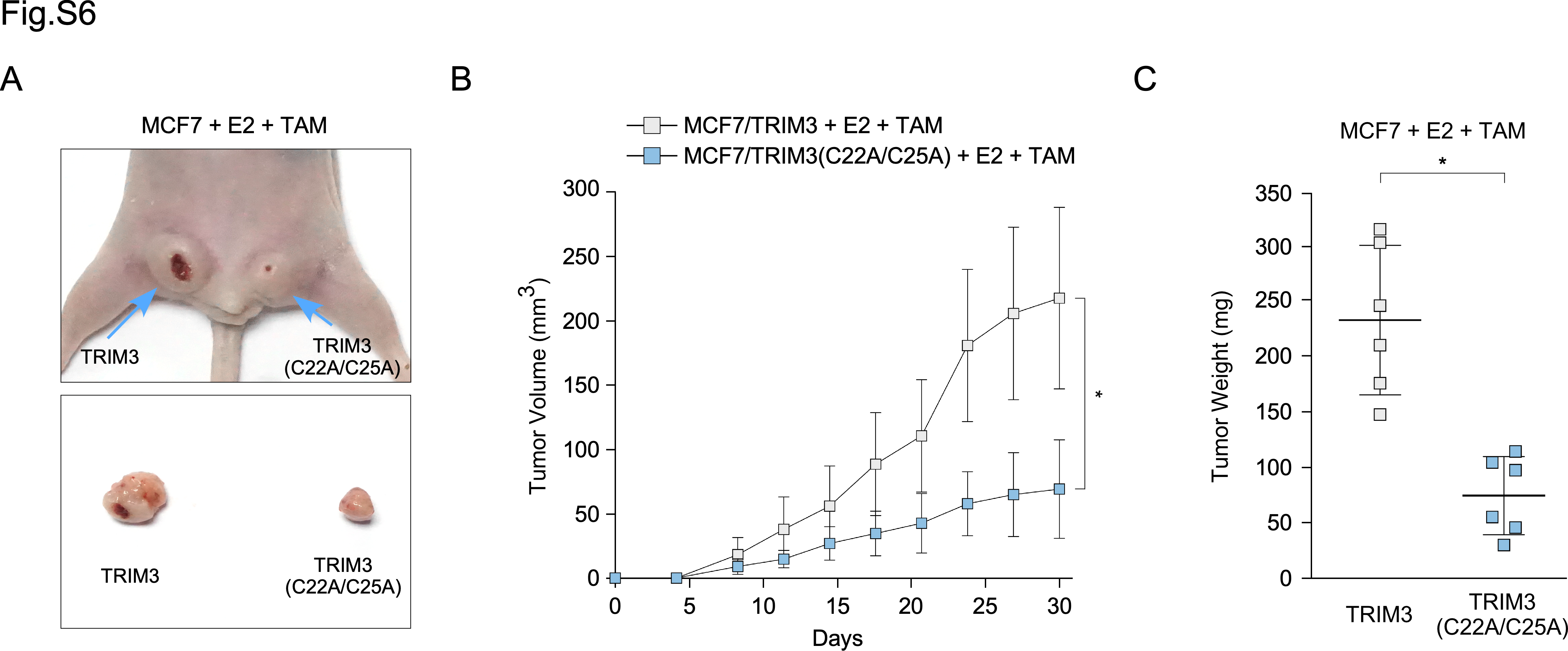

Supplement: Supplementary file 7 — Supplementary Fig. S6 [file 41389_2021_350_MOESM7_ESM.tif]
